# Factors affecting Kaposi’s sarcoma-associated herpesvirus transmission in rural Ugandan households, a longitudinal study

**DOI:** 10.21203/rs.3.rs-4855275/v1

**Published:** 2024-09-10

**Authors:** Katherine R. Sabourin, Vickie A. Marshall, Will Eaton, Beatrice Kimono, Joseph Mugisha, Wendell J. Miley, Nazzarena Labo, Gabriela Samayoa-Reyes, Denise Whitby, Rosemary Rochford, Robert Newton

**Affiliations:** University of Cincinnati; Leidos Frederick; Tulane University; MRC/UVRI and LSHTM Uganda Research Unit; MRC/UVRI and LSHTM Uganda Research Unit; Leidos Frederick; Leidos Frederick; University of Colorado; Leidos Frederick; University of Colorado; University of York

**Keywords:** Kaposi’s sarcoma-associated herpesvirus, KSHV, HHV-8, HIV, Longitudinal Follow-up, Transmission, Shedding, Saliva, sub-Saharan Africa, Household cohort

## Abstract

**Background:**

We report the impact of HIV infection within a household on oral Kaposi’s sarcoma-associated herpesvirus (KSHV) shedding.

**Methods:**

We enrolled 469 individuals from 90 households. Mouthwash rinse samples collected at three monthly visits, were analyzed for KSHV DNA using quantitative polymerase chain reaction (qPCR). Generalized linear mixed effects logistic models were applied to analyze factors associated with KSHV ever shedding, and among shedders, always versus intermittent shedding. Linear mixed effects models were applied to models of KSHV viral loads. Intraclass correlation coefficients (ICCs) were calculated to assess the contribution of household-level factors to variations in shedding probabilities. Hotspot analyses of geospatial feature clusters were calculated using Getis-Ord Gi* statistic and visualized using inverse distance weighted interpolation.

**Results:**

Analyses included 340 KSHV seropositive individuals, aged 3 + years, with qPCR results from 89 households. Forty households had 1 + persons living with HIV (PLWH), while 49 had none. Among participants, 149(44%) were KSHV ever shedders. Of 140 who shed KSHV at two or more visits, 34(24%) were always shedders. Increasing number of KSHV seropositive household members was significantly associated with ever shedding [Odds ratio(OR) (95% Confidence Interval(95%CI)):1.14(1.03,1.26);p = 0.013]. Among KSHV shedders, a statistically significant age-related trend was identified with 10–19 years being more likely to be always shedders (type III test p = 0.039) and to have higher viral loads (type III test p = 0.027). In addition, higher viral loads were significantly associated with increasing number of household members [coefficient(95%CI):0.06(0.01,0.12);p = 0.042], increasing number of KSHV seropositive members [coefficient(95%CI):0.08(0.01,0.15);p = 0.021], and living in households with 1 + PLWH [coefficient(95%CI):0.51(0.04,0.98);p = 0.033]. Always shedders exhibited higher viral loads than intermittent shedders [coefficient(95%CI):1.62(1.19,2.05);p < 0.001], and viral loads increased with the number of visits where KSHV DNA was detected in saliva (type III test p < 0.001). Household-level factors attributed for 19% of the variability in KSHV shedding (ICC:0.191;p = 0.010). Geospatial analysis indicated overlapping hotspots of households with more KSHV seropositive individuals and KSHV shedders, distinct from areas where PLWH were clustered.

**Discussion:**

KSHV oral shedding is influenced by multiple factors at the individual, household, and regional levels. To mitigate ongoing KSHV transmission a comprehensive understanding of factors contributing to oral KSHV reactivation and transmission within households is needed.

## Background

Transmission of Kaposi’s sarcoma-associated herpesvirus (KSHV), the causative agent of Kaposi’s sarcoma (KS), occurs primarily through oral fluids (1, 2). Though different demographic, social, and environmental factors have been linked to KSHV reactivation and shedding in the oral cavity, shedding dynamics over time and the factors that affect viral transmission, especially in sub-Saharan Africa where KSHV seroprevalence is highest, are still relatively unknown. Initial KSHV infection in sub-Saharan Africa occurs during childhood, so KSHV shedding within a household may be an important source of continued exposure. Previously, a strong dependence on being KSHV seropositive was identified between mother-child pairs and between sibling pairs from the Brazilian Amazon (3) and detection of KSHV DNA in oral fluids was associated with household density in Uganda (4). However, the dynamics and risk factors within a household that support transmission have still not been adequately identified.

HIV infection is strongly associated with KSHV seropositivity and KS development; however, its effect on KSHV shedding remains poorly understood. Prior cross sectional and case control studies have reported increased likelihoods of KSHV shedding among PLWH (5–7) while others have not (8, 9). While longitudinal studies reported no difference in KSHV shedding by HIV status (1, 10–13), these may have been limited by small sample sizes. Among individuals with KSHV detected in oral fluids, KSHV viral loads have also been reported to be higher among PLWH in some studies (10, 14) though others have reported no associations (1, 6, 7). Because KSHV shedding and serostatus fluctuate over time (10–12, 15–17), studies of HIV status and KSHV shedding require a longitudinal design, and a large enough study population to ensure adequate power to identify the effects of HIV on oral KSHV reactivation. In addition, the role of HIV infection within families and how it may contribute to increases in viral shedding over time and, consequently, transmission has not been adequately explored.

HIV prevalence in the Ugandan-based general population cohort (GPC) has remained relatively stable at 6–7% since 2005 (18, 19). We designed a longitudinal study of household clusters selected from the GPC to define KSHV viral shedding profiles over time, as these are known to fluctuate in a single individual(20), as well as determine the role of HIV within a household on increased risk of transmission and transmission dynamics of KSHV.

## Methods

### Setting

The general population cohort (GPC) is an open community cohort based in Kyamulibwa subcounty in Kalungu District in southwestern Uganda. The GPC, first established in 1989 to study the epidemiology of HIV in a rural African population, consists of 22,000 individuals from 25 neighboring villages (18). KSHV seroprevalence in this region is the highest reported at over 90% (21) and HIV prevalence has remained stable at 6–7% (18, 19). Regular annual census and bi-annual survey rounds are completed. Within the census GPS coordinates and other census data are obtained from the households. Data on HIV status are obtained from the survey rounds.

### Study Design

Between August-October 2021, we enrolled members from 90 households in a longitudinal cohort study. Households were pre-identified using HIV statuses collected from GPC survey data and included 45 households with at least one member living with HIV (PLWH) and 45 households with no members living with HIV. Anyone staying in the home at least 50% of the time was welcome to participate, regardless of age or sex. After enrollment, two follow-up visits at one month apart for a total of three visits were completed. Ethical approval was obtained by the Uganda Virus Research Institute Research Ethics Committee (UVRI-REC), Uganda National Council for Science and Technology (UNCST), and Colorado Multiple Institutional Review Board (COMIRB). All participants provided written/thumb printed informed consent. Parent/guardian consent was obtained for participants aged < 18 years. Assent was obtained from children aged 8–17 years. Procedures were completed in accordance with the ethical standards of the Helsinki Declaration.

### Data collected

At each visit a questionnaire was administered by the field team which collected data on sex, age, and household size. Socioeconomic statuses were derived from variables collected on the characteristics of the household’s main house (roof, walls, etc), whether owned by household members, and whether household members owned any building for rent (house, shop, kiosk, etc). Principal components were calculated using the covariance matrix households classified into five quantiles. If individuals had been ill in the last month, they were asked to provide information on clinical symptoms, whether they accessed care, type of care accessed, treatment given for their illness, and final diagnosis.

### HIV determination

At enrollment, data was collected by self-report on each individual’s HIV infection status, years since HIV diagnosis, and current anti-retroviral therapy (ART) use, if applicable. For a subset of individuals, we were able to confirm HIV status using previously completed study data. HIV seropositive individuals were those who self-reported as HIV positive or whose HIV status was confirmed using previously collected GPC census or other study data.

All others were considered HIV negative. Household HIV status was reassessed and re-categorized based on identified individual HIV status.

### Sample processing

Up to 5 ml of venous blood was collected in EDTA tubes for each individual at baseline, 1.5 ml whole blood was removed and stored for subsequent DNA extraction. The remaining blood was centrifuged at 10,000 rpm for 5 minutes at room temperature, and plasma removed and stored for antibody testing. At enrollment and each follow-up study participants rinsed their mouth with 2.5mL Listerine mouthwash. After collection, up to 2 ml of oral fluid was transferred and spun at room temperature at 1500×g for 10 minutes. Supernatant was removed from the cell pellet. The cell pellets were used for subsequent analysis. All blood and oral fluid samples were stored at −80°C until further analysis.

### KSHV serology

Plasma samples collected at baseline were tested by multiplex bead-based assay for detection of anti-KSHV and anti-EBV antibodies as previously described (22). The panel included 25 KSHV recombinant proteins (K3, K5, K6, K8, K11, K8.1, K10.5, K14, ORF11, ORF18, ORF19, ORF2, ORF20, ORF25, ORF26, ORF33, ORF34, ORF35, ORF37, ORF38, ORF43, ORF44, ORF45, ORF50, ORF52, ORF55, ORF59, ORF6, ORF60, ORF61, ORF63, ORF65, ORF69, ORF72, ORF73) and three EBV proteins (EBNA-1, VCA, and EA). Median fluorescence intensity (MFI) across all counted beads was computed by subtracting background fluorescence. Negative controls included healthy US-based adult blood donors at low risk of KSHV. Positive controls included US-based adults with active or history of KSHV-associated disease and/or KSHV DNA detected in peripheral blood mononuclear cells (PBMC). Receiver operating curves or a parametric distribution using negative and positive control sera were used to identify cut-offs (22). Individuals were identified as KSHV seropositive if IgG to any of the 25 KSHV antigens were detected in plasma. EBV seropositivity was defined as detection of anti-EBV IgG antibodies to VCA or EBNA-1.

### KSHV shedding

Mouthwash rinse samples were tested for detection of KSHV DNA using qPCR as previously described (23). Briefly, the Qiagen blood mini kit (Qiagen, Valencia, CA) was used to extract DNA from oral fluid cell pellets and the DNA was tested using primers specific to the KSHV K6 gene region and the human endogenous retrovirus 3 (ERV-3) gene which is used for cell quantification (24). Samples were run in triplicate in both assays and the average values of the independent reactions calculated. KSHV viral load was reported as copies per million cell equivalents. Individuals were identified as KSHV shedders if KSHV DNA was detected in oral fluids at any timepoint and non-shedders if not. Among KSHV shedders, we categorized individuals as either always shedders, if KSHV DNA was detected in oral fluids in all samples collected, or intermittent shedders, if KSHV DNA was detected at least once but not in all samples. Individuals with only one visit were included in the shedder vs non-shedder analysis but excluded from the analyses of always vs intermittent shedders. KSHV viral loads were log transformed prior to analyses.

### Statistical Analyses

For analyses of risk factors for KSHV shedders we excluded individuals who were KSHV seronegative, who did not have oral fluids tested for KSHV DNA by qPCR, or who were under three years of age due to difficulties with collecting valid mouthwash samples from younger children. Descriptive statistics were used to describe individuals included in final analyses. Generalized linear mixed logistic regression model with a specified binomial distribution and included logit link function were used to model risk factors for being an ever vs never shedder and among shedders with 2 + visits, of being an always vs intermittent shedder using PROC GLIMMIX in SAS. Models included a random intercept and random slope for household effects. To model risk factors for increased levels of KSHV viral loads among shedders, we applied linear mixed effects models including a random intercept and random slope for individual effects and an unstructured covariance structure. Type III tests of fixed effects were used to identify if trends were statistically significant. Confounding variables identified *a priori* were included in adjusted analyses if they were associated with both the exposure and outcome by p < 0.02 and changed the crude estimate by 10+%. If no variables met these criteria, then crude estimates were reported. To determine the amount of total variation in the probability of being an ever vs never KSHV shedder, and among KSHV shedders of being an always vs intermittent shedder that is accounted for by household we calculated the intraclass correlation coefficient (ICC) (25). P-values < 0.05 considered statistically significant. Models of risk factors were completed using SAS 9.4 [SAS Institute In, Cary, NC].

### Geospatial Analysis

Descriptive maps and spatial analyses were completed in R version 4.3.3. Hot-spot analyses generating local Getis-Ord G_i_*statistics using ‘spdep’ R package (26–29) were performed to detect statistically significant clusters of high and low feature values within the study area, which are unlikely the result of random chance. Inverse distance weighted (IDW), deterministic spatial interpolation methods were performed using the ‘gstat’ R package (30) to visualize feature hot and cold spot distribution (29). Hot-spot analysis results are presented as a Gi* statistic, which is represented as a Z-score, where higher values signify a larger clustering intensity, while the (positive or negative) direction indicate high or low value clusters, respectively. An associated p-value indicating statistically significant results is also provided. R code is available on github repository (31).

## Results

### Participant inclusion

We enrolled 469 participants of all ages from 90 households. Of those we excluded 129 (28%) individuals who either had no KSHV serology results (n = 28, 6%), were KSHV seronegative (n = 75, 16%), did not have oral fluids tested for KSHV DNA by qPCR (n = 22, 5%), or were under three years of age due to difficulties with collecting valid mouthwash samples from younger children (n = 4, < 1%). The majority (n = 56, 75%) of those who were KSHV seronegative were under 10 years of age. This resulted in the inclusion of 340 KSHV seropositive participants from 89 households. We confirmed the status of at least one PLWH in 40 of the included households (positive household HIV status), leaving 49 households defined as having no PLWH.

### Household and participant characteristics

Household member size ranged from 1 to 16, with an average of 5.2 (standard deviation: 2.9) individuals per home. In one household, consisting of two members only, no KSHV seropositive individuals were identified, despite the high sensitivity of the multiplex assay. Of the 89 households included in our analysis, the number of KSHV seropositive members ranged from 1 to 14 with an average of 4.2 (2.7) per home. The proportion of KSHV seropositive members ranged from 0.2 to 1.0 per home with an average of 0.8(0.2).

Of the 340 KSHV seropositive participants included in our final analyses, 25 (7%) completed one visit, 57 (17%) two visits, and 258 (76%) completed all three visits. Half of participants were female (54%), and half were children under 14 years of age (49%). Of included participants, 14% were PLWH, while HIV serostatus was missing for 27%. Among individuals who self-reported an HIV infection, all stated they were currently on ART and a median of seven years had passed from initial HIV diagnosis. Nearly all participants were classified as EBV seropositive.

Participants from households with at least one PLWH compared to households with none were more likely to be over 40 years of age (30% vs 14%), and more often classified into the fourth or higher SES quintile (68% vs 29%). Fewer individuals from households with a PLWH reported being sick at the time of visit compared to households with no PLWH (8% vs 26%) [Table 1].

### Ever vs never KSHV shedders

Of the 340 participants, 149 (44%) were identified as ever shedders. Among ever shedders, 59% had one visit with shedding, 22% had two, and 19% had KSHV DNA detected at all three visits [Table 1]. This was similar when restricted to 258 individuals with all three visits, of whom 125 (48%) were identified as ever shedders (1 shedding visit 56%, two shedding visits 22%, all three visits 22%).

Approximately 19% (ICC = 0.191) of variability in shedding was accounted for by households in the study while 81% of the variability in shedding was accounted for by individual or other factors outside of the household. There was a statistically significant amount of variability in the log odds of being an ever shedder between the households in our sample [*ττ*00 = .7746; p = 0.010]. Higher odds of shedding were statistically significantly associated with an increasing number of KSHV seropositive household members (Odds ratio (OR): 1.14 (95% Confidence interval, CI: 1.03–1.26; p = 0.013) and with an increasing number of household members in general (OR: 1.10, (95% CI, 1.00,1.21; p = 0.051). There were no other statistically significant risk factors for KSHV shedding at the individual or the household level including the individual’s HIV infection [Table 2].

### Always vs intermittent KSHV shedders

Of the 149 identified KSHV shedders, we included 140 in analyses of always shedders [n = 34 (24%)] compared to intermittent shedders, [n = 106 (76%)]. The variability between households in the log odds of being an always shedder was not statistically significant [*ττ*00 = .0221; p = 0.474] and only < 1% of variability in always shedding was accounted for by households in the study (ICC = 0.007) meaning that 99% of the variability in being an always shedder is accounted for by individual or other factors. We identified a statistically significant trend between age and the likelihood of being an always shedder, with 10–19-year-olds having the highest likelihood (Type III p = 0.039) [Table 3].

### KSHV viral load in oral fluids

We found a similar statistically significant trend in KSHV viral loads based on age, with higher average KSHV viral loads identified in 10–19-year-olds, after adjustment for number of household members and household HIV status (Type III p = 0.027). On average, higher KSHV viral loads were identified in individuals living with more household members [coefficient = 0.06, 95% confidence interval (95%CI):0.01,0.02, p = 0.042] and living with more KSHV seropositive household members after adjustment for household HIV status [coefficient = 0.08, 95%CI:0.01,0.15;p = 0.021], and among those living in a household with at least one PLWH, after adjustment for number of household members [coefficient = 0.51, 95%CI:0.04,0.98, p = 0.033]. KSHV viral loads in oral fluids were also higher on average in always vs intermittent shedders [coefficient = 1.62, 95%CI:1.19,2.05; p < 0.001] and with increasing number of samples with KSHV DNA detected [Table 4]. When restricted to individuals with all three visits, the direction and magnitude of the association of increasing number of samples with KSHV DNA detected and KSHV viral loads did not change.

### Descriptive maps and hot spot analyses

**Figure 1:**
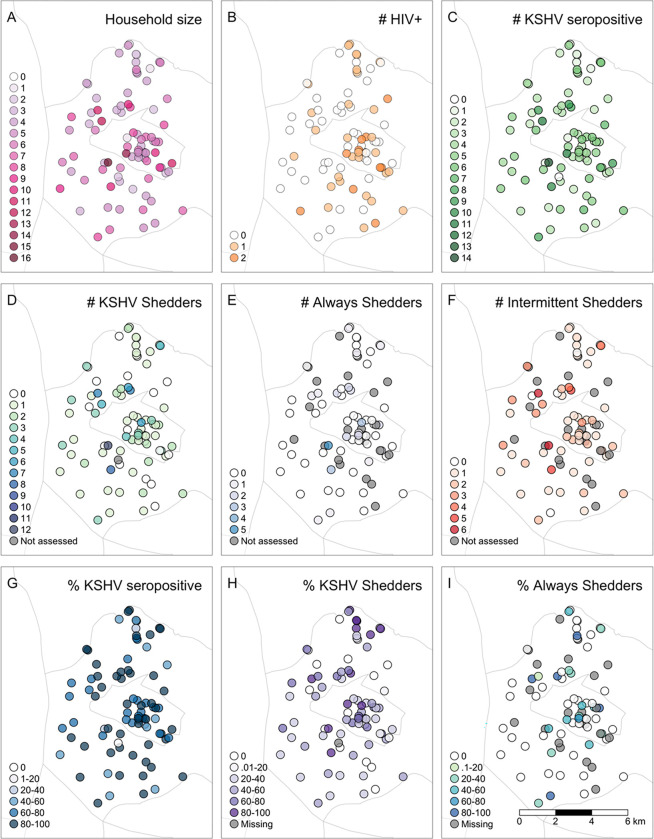
Geospatial Distribution of Households. A) Number of individuals per household B) Number of PLWH per household; C) Number of KSHV seropositive individuals per household; D) Number of KSHV shedders per household; E) Number of KSHV shedders who always had KSHV detected in saliva per household (always shedders); F) Number of KSHV shedders who had at least one visit with and one visit without KSHV detected in saliva per household (intermittent shedders); G) Proportion of a household who are KSHV seropositive; H) Proportion of shedders among KSHV seropositive individuals tested in a household; I) Proportion of household shedders who were always shedders.

We provide a geospatial distribution of households by size, HIV and KSHV serostatus, and KSHV shedding status in Fig. 1. Cluster analyses identified larger household sizes in the west and in the center and coldspots of lower household size in the north of the region. These hotspots were similar to the location of hotspots of KSHV seropositive individuals per household found in the west and center but no statistically significant coldspots of KSHV seropositivity were identified. Households with higher numbers of PLWH were identified in the southeast while those with lower numbers of PLWH and with higher numbers of KSHV seropositive individuals were identified in the west. There was no spatial overlap of hotspots of PLWH per household with hotspots of KSHV seropositive individuals per household.

**Figure 2:**
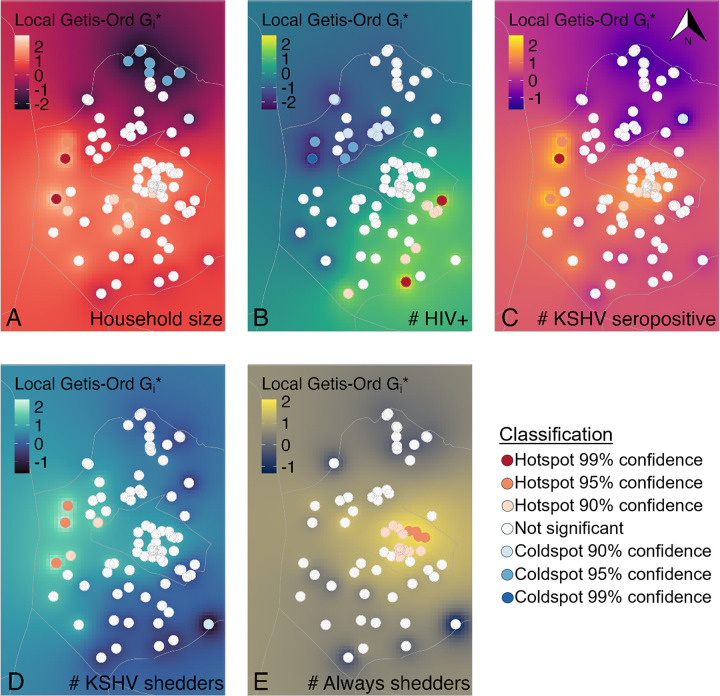
Hotspot analysis of HIV, KSHV seropositivity, and KSHV shedding by geographic location. Filled circles represent statistically significant hotspots and coldspots. From Left to Right: A) Number of individuals per household; B) Number of PLWH per household; C) Number of KSHV seropositive individuals per household; D) Number of KSHV shedders per household; E) Number of KSHV shedders who always had KSHV detected in saliva per household.

Five statistically significant hotspots of higher numbers of KSHV shedders per household were located in the west of the GPC and these overlapped spatially with three households that were statistically significant hotspots for both larger household size and higher number of KSHV seropositive individuals per household. Approximately two hotspot locations of KSHV seropositive individuals and KSHV shedders overlapped spatially with two statistically significant cold spots of individuals with HIV per household. No statistically significant cold spots of lower numbers of KHSV shedders per household were detected. Among households with at least one KSHV shedder, statistically significant hotspots of higher numbers of always shedders clustered in the middle of the region, while no coldspots were identified [Figure 2].

## Discussion

In this longitudinal study of rural Uganda households, we found that living with at least one PLWH, though not associated with the probability of KSHV shedding, was associated with higher KSHV viral loads among KSHV shedders. The number of KSHV seropositive residents in a household was associated with KSHV shedding and with increased viral loads among shedders. We also identified a moderate variability in being a KSHV ever shedder that can be accounted for at the household level. In addition, both KSHV shedding frequency and viral loads in the oral fluids appeared to be age dependent, and individuals with more frequent shedding had higher viral loads.

The variability in being an ever shedder accounted for by household was moderate, at 19%. This variability, as measured by intraclass correlation analysis, likely stems from household-related factors that promote KSHV reactivation in the oral cavity. Our work in another Ugandan cohort identified higher KSHV viral loads in the oral fluids of children with helminthiasis and an inverse association between KSHV shedding and anti-malaria antibody levels (32), suggesting that co-infections, which may be common within a household, may play a role in oral KSHV reactivation. Prior studies in this cohort and in a pediatric sickle cell study, however, found no associations between oral KSHV shedding and parasitic infections, including malaria (4, 33). Unfortunately, our study lacked data on individual infections, precluding adjustment for these factors.

We did find a statistically significant association between the number of KSHV seropositive household members and the probability of ever shedding KSHV. Previous studies in Zambia reported an elevated risk of KSHV infection in children with increasing number of KSHV seropositive household members (34) but did not examine the likelihood of KSHV shedding among those already seropositive. However, we also found an effect of household size in general. These findings suggest that while household-specific factors may contribute to KSHV shedding, individual-level factors primarily drive oral KSHV reactivation and shedding probability.

Among KSHV shedders, we observed a statistically significant age-related trend, with individuals aged 10–19 being more likely to be always shedders, with higher viral loads. These findings align with previous reports from Uganda, indicating age-dependent variations in KSHV shedding dynamics. Specifically, that KSHV viral loads in oral fluids were highest in 6–12-year-old shedders decreasing with age afterwards (33) decreasing with age afterwards and that detection of KSHV DNA was twice as likely in children compared to their mothers (32). These studies also reported a higher likelihood of KSHV shedding and higher viral loads in males (32, 33) though we did not find this same association by sex. Nevertheless, our findings underscore the significance of age in ongoing KSHV transmission, particularly the possible contribution of children within households.

Among KSHV shedders, individuals living with at least one PLWH exhibited higher viral loads.

Previous studies have reported higher KSHV seropositivity among children of women living with HIV/AIDS (WLHA)(35–37), potentially indicative of a role of HIV infection in promoting reactivation of KSHV in the oral cavity and supporting transmission within a household. We did not however identify associations between individual HIV status and likelihood of KSHV shedding or viral loads. Inconsistent results have been reported regarding the effect of HIV infection on KSHV shedding (5–9, 14, 38) though most previous longitudinal studies reported no relation between KSHV shedding and HIV status (1, 10–13, 17, 39, 40). Among KSHV shedders, the association between HIV infection and KHSV viral loads in PLWH have also been inconsistently observed (1, 6, 10, 14) suggesting a complex interaction may exist between HIV and oral KSHV reactivation.

Additionally, we found that KSHV viral loads were higher among individuals who consistently shed the virus compared to those who shed intermittently, with viral loads increasing with the frequency of shedding episodes. This corroborates findings from previous studies in Kenya and an analysis across multiple regions including the US, Peru, Cameroon, Uganda, and Kenya, which indicated a positive correlation between shedding frequency and viral load levels (11, 12). These findings are also supported by a study in North America reporting that past shedding predicted future shedding occurrences and corresponding viral load levels in saliva (41). The factors influencing shedding frequency and their predictive value for viral loads require additional study.

Our geospatial analysis revealed overlapping hotspots of KSHV seropositivity and shedding within households, particularly in the western region of the study area. These hotspots differed from hotspots of HIV infection. The correspondence between hotspots of KSHV seropositivity and shedding with hotspots of larger household size in the west suggests an association between KSHV seropositivity and shedding and proximity to larger household size in increased housing density settings. The number of individuals per household tended to be low in the north and was confirmed by hotspot analyses, revealing statistically significant coldspots of household size. Therefore, statistically significant spatial clusters were not expected in this region. Among households with at least one KSHV shedder we did see clustering of hotspots towards the center of the region which differed from those identified for shedding. The observed spatial clustering suggests regional-level exposures common to households that may support KSHV transmission and continued infection, warranting further investigation.

This study was nested within an established well characterized population-based cohort, which minimizes heterogeneity between households with and without PLWH. However, it did have limitations including the reliance on self-reported and previously collected data to identify PLWH, potentially leading to an underestimation of PLWH in our cohort. Additionally, lack of data on CD4 counts or viral loads for PLWH limited our ability to explore their impact on KSHV shedding dynamics. Though we were able to follow participants longitudinally, our study did include a relatively short follow-up time. Risk factors for long term shedding may differ from those for shedding over shorter periods of time and should be examined in both contexts.

## Conclusions

Household levels factors such as living with a PLWH, the number of KSHV seropositive individuals in a home, and other undefined household level factors play a role in oral KSHV shedding. However, it appears that individual factors, predominately an individual’s age, are more significant predictors of oral KSHV shedding. Overall, the factors leading to KSHV reactivation in the oral cavity are multifactorial and include individual, household, and regional level factors that require further identification. A thorough exploration of the factors contributing to oral KSHV reactivation and sustained transmission within households in sub-Saharan Africa, would contribute to reducing the burden of KS where it is highest.

## Figures and Tables

**Table 1:** Characteristics of KSHV Seropositive Participants by Household HIV Status

	Household HIV		
	No PLWH (49 households, n = 195 participants)	1 + PLWH (40 households, n = 145 participants)	Total (89 households, n = 340 participants)
Characteristic	n(%)	n(%)	n(%)
**Sex** – Female vs Male	103(52.8)	79(54.5)	182(53.5)
**Age (years)** – Mean [std]	20.7[18.2]	25.8[19.3]	22.9[18.8]
**Age (years)**			
1–4 years	17(8.7)	8(5.5)	25(7.4)
5–9 years	44(22.6)	25(17.2)	69(20.3)
10–14 years	41(21.0)	33(22.8)	74(21.8)
15–19 years	28(14.4)	11(7.6)	39(11.5)
20–29 years	17(8.7)	10(6.9)	27(7.9)
30–39 years	20(10.3)	15(10.3)	35(10.3)
40 + years	28(14.4)	43(29.7)	71(20.9)
**Socioeconomic Status Quintiles**			
Highest	6(3.1)	39(26.9)	45(13.2)
Fourth	51(26.2)	59(40.7)	110(32.4)
Middle	50(25.6)	18(12.4)	68(20)
Second	22(11.3)	19(13.1)	41(12.1)
Lowest	4(2.1)	10(6.9)	14(4.1)
**Number of Household Members** – Mean per household [std]	5.7[2.9]	4.7[2.7]	5.2[2.9]
**Number of KSHV Seropositive Household Members** – Mean per household[std]	4.4[2.9]	4.0[2.4]	4.2[2.7]
**Sick at time of visit** – yes vs no	51(26.2)	12(8.3)	63(18.5)
**HIV Serostatus**			
Seropositive	0(0.0)	49(33.8)	49(14.4)
Missing	61(31.3)	32(22.1)	93(27.4)
**ART Status** – currently on ART vs. Not (n = 39)	-	39 (100%)	39 (100%)
**Years since HIV diagnosis** (n = 39) – Median [range]	-	9[0,27]	9[0,27]
**EBV Status** – Seropositive vs Seronegative	194(99.5)	145(100.0)	339(99.7)
**Total Study Visits**			
1	13(6.7)	12(8.3)	25(7.4)
2	35(17.9)	22(15.2)	57(16.8)
3	147(75.4)	111(76.6)	258(75.9)
**KSHV Shedders** – ever vs never	85(43.6)	64(44.1)	149(43.8)
**Among Shedders, Number of Visits with KSHV Shedding**			
1	53(62.4)	35(54.7)	88(59.1)
2	19(22.4)	14(21.9)	33(22.1)
3	13(15.3)	15(23.4)	28(18.8)

Acronyms Used: Antiretroviral therapy (ART), Epstein Barr Virus (EBV), Kaposi’s sarcoma-associated herpesvirus (KSHV), Human Immunodeficiency Virus (HIV), People living with HIV (PLWH)

All variables missing < 2% data except where specified.

HIV seropositive individuals were those who self-reported as HIV positive or whose HIV status was confirmed using previously collected GPC census or other study data.

Years since HIV diagnosis and ART status were available for those who self-reported their HIV status (n = 39).

EBV seropositivity was defined as detection of anti-EBV antibodies to VCA or EBNA-1.

**Table 2:** Risk Factor Associations with KSHV Ever vs Never Shedding (n = 340)

Risk Factors	Crude Estimates		Adjusted Estimates	
	OR (95% CI)	p	OR (95% CI)	p
**Sex** – Female vs Male	0.77(0.47,1.27)	0.303	-	
**Age (years)** – continuous	1.00(0.99,1.01)	0.763	-	
**Age (years)**		0.633		
1–4 years	REF		-	
5–9 years	1.16(0.39,3.41)	0.792	-	
10–14 years	1.62(0.56,4.71)	0.375	-	
15–19 years	1.94(0.59,6.35)	0.275	-	
20–29 years	1.49(0.41,5.39)	0.541	-	
30–39 years	0.80(0.24,2.69)	0.711	-	
40 + years	1.08(0.36,3.18)	0.896	-	
**Socioeconomic Status Quintiles** ^a^		0.238		0.264
Highest	REF		REF	
Fourth	1.29(0.58,2.85)	0.528	1.48(0.68,3.20)	0.321
Middle	0.72(0.30,1.75)	0.471	0.89(0.37,2.16)	0.799
Second	1.22(0.45,3.36)	0.693	1.53(0.56,4.14)	0.405
Lowest	0.18(0.03,1.16)	0.071	0.25(0.04,1.56)	0.136
**Number of Household Members** – continuous	1.10(1.00,1.21)	0.051	-	
**Number of KSHV Seropositive Household Members** – continuous	1.14(1.03,1.26)	**0.013** *	-	
**Sick at time of visit** – yes vs no	1.07(0.48,2.40)	0.870	-	
**EBV Serostatus** – seropositive vs seronegative	0.01(< 0.01,>999.9)	0.858	-	
**Individual HIV Serostatus** – seropositive vs seronegative	0.62(0.29,1.35)	0.228	-	
**Household HIV Status** – At least one PLWH vs No PLWH^a^	1.10(0.58,2.06)	0.774	1.22(0.66,2.24)	0.529
**Total Study Visits**				
1	REF		-	
2	0.61(0.20,1.88)	0.387	-	
3	1.54(0.58,4.06)	0.384	-	

Acronyms Used: Antiretroviral therapy (ART), Epstein Barr Virus (EBV), Kaposi’s sarcoma-associated herpesvirus (KSHV), Human Immunodeficiency Virus (HIV), People living with HIV (PLWH)

All variables missing < 2% data except where specified.

HIV seropositive individuals were those who self-reported as HIV positive or whose HIV status was confirmed using previously collected GPC census or other study data.

Years since HIV diagnosis and ART status were available for those who self-reported their HIV status (n = 39).

KSHV seropositivity was defined as detection of anti-KSHV IgG antibodies to K3, K5, K6, K8, K11, K8.1, K10.5, K14, ORF11, ORF18, ORF19, ORF2, ORF20, ORF25, ORF26, ORF33, ORF34, ORF35, ORF37, ORF38, ORF43, ORF44, ORF45, ORF50, ORF52, ORF55, ORF59, ORF6, ORF60, ORF61, ORF63, ORF65, ORF69, ORF72, or ORF73.

EBV seropositivity was defined as detection of anti-EBV IgG antibodies to VCA or EBNA-1.

* p-values < 0.05 considered statistically significant.

All models included a random intercept and random slope for household.

a SES and Household HIV Adjusted for number of household members (continuous)

**Table 3:** Risk Factors Associations with KSHV Always vs Intermittent Shedders (n = 140 shedders)

Risk Factors	Crude Estimates		Adjusted Estimates	
	OR (95% CI)	p	OR (95% CI)	p
**Sex** – Female vs Male^a^	0.71(0.32,1.55)	0.381	0.80(0.33,1.95)	0.618
**Age (years)** – continuous	0.99(0.96,1.01)	0.210	-	
**Age (years)**		0.039*		
1–4 years	REF		-	
5–9 years	0.98(0.08,11.55)	0.984	-	
10–14 years	5.85(0.63,54.60)	0.119	-	
15–19 years	5.90(0.57,60.98)	0.134	-	
20–29 years	1.98(0.14,28.56)	0.610	-	
30–39 years	0.90(0.04,18.01)	0.941	-	
40 + years	0.97(0.08,11.56)	0.980	-	
**Socioeconomic Status Quintiles**		0.915		
Highest	REF		-	
Fourth	0.93(0.34,2.55)	0.881	-	
Middle	1.28(0.38,4.36)	0.688	-	
Second	1.01(0.25,4.13)	0.989	-	
Lowest	3.35(0.17,67.67)	0.426	-	
**Number of Household Members** – continuous	Model did not converge		-	
**Number of Household KSHV Seropositive Members** – continuous	1.11(0.99,1.24)	0.076	-	
**Sick at time of visit** – yes vs no^a^	0.91(0.29,2.79)	0.861	0.76(0.22,2.62)	0.662
**EBV Serostatus** – seropositive vs seronegative^a^	> 999.9(< 0.01,>999.9)	0.985	> 999.9(< 0.01,>999.9)	0.995
**Individual HIV Serostatus** – positive vs negative^a^	0.91(0.17,4.92)	0.908	3.48(0.30,40.52)	0.310
**Household HIV Status** – At least one PLWH vs No PLWH	1.41(0.64,3.11)	0.391	-	
**Total Study Visits**				
2	REF		-	
3	0.42(0.13,1.38)	0.152	-	

Acronyms Used: Antiretroviral therapy (ART), Epstein Barr Virus (EBV), Kaposi's sarcoma-associated herpesvirus (KSHV), Human Immunodeficiency Virus (HIV), People living with HIV (PLWH)

KSHV seropositivity was defined as detection of anti-KSHV IgG antibodies to K3, K5, K6, K8, K11, K8.1, K10.5, K14, ORF11, ORF18, ORF19, ORF2, ORF20, ORF25, ORF26, ORF33, ORF34, ORF35, ORF37, ORF38, ORF43, ORF44, ORF45, ORF50, ORF52, ORF55, ORF59, ORF6, ORF60, ORF61, ORF63, ORF65, ORF69, ORF72, or ORF73.

EBV seropositivity was defined as detection of anti-EBV IgG antibodies to VCA or EBNA-1. p-values < 0.05 considered statistically significant.

All models included a random intercept and random slope for household.

a Adjusted for age (categorical)

**Table 4:** Associations between Risk Factors and log-transformed KSHV viral loads among KSHV Shedders (n = 149).

	Crude Estimates		Adjusted Estimates	
Risk Factors	Beta(95%CI)	p-value	Beta(95%CI)	value
**Sex** – Female vs Male^a,b^	−0.17(−0.64,0.29)	0.461	−0.07(−0.55,0.41)	0.778
**Age (years)** – continuous^b^	−0.01(−0.02,0.01)	0.168	−0.01(−0.02,0.01)	0.260
**Age (years)** ^b,c^		**0.046** *		**0.027** *
1–4 years	REF		REF	
5–9 years	−0.26(−1.27,0.75)	0.612	−0.39(−1.39,0.61)	0.443
10–14 years	0.65(−0.34,1.63)	0.197	0.61(−0.36,1.58)	0.213
15–19 years	0.36(−0.72,1.45)	0.510	0.36(−0.71,1.43)	0.506
20–29 years	−0.11(−1.27,1.05)	0.852	−0.19(−1.33,0.95)	0.743
30–39 years	−0.53(−1.76,0.70)	0.397	−0.43(−1.64,0.78)	0.485
40 + years	−0.31(−1.32,0.71)	0.555	−0.37(−1.37,0.62)	0.460
**Socioeconomic Status Quintiles** ^b,c^		0.917		0.877
Highest	REF		REF	
Fourth	0.23(−0.35,0.80)	0.434	0.32(−0.28,0.91)	0.294
Middle	0.02(−0.69,0.72)	0.966	0.25(−0.48,0.97)	0.500
Second	−0.01(−0.77,0.77)	0.996	0.26(−0.55,1.07)	0.525
Lowest	0.42(−1.46,2.29)	0.661	0.41(−1.47,2.29)	0.666
**Number of Household Members** – continuous^c^	0.05(−0.01,0.11)	0.113	0.06(0.01,0.12)	**0.042** *
**Number of KSHV Seropositive Household Members** – continuous^c^	0.07(0.01,0.13)	**0.036** *	0.08(0.01,0.15)	**0.021** *
**Sick at time of visit** – yes vs no,^c^	−0.42(−1.04,0.20)	0.182	−0.35(−1.01,0.31)	0.291
**EBV Status** – Seropositive vs Seronegative	0.60(−1.98,3.19)	0.645	--	
**Individual HIV Serostatus** – positive vs negative^a,b^	0.06(−0.62,0.74)	0.860	0.67(−0.1,1.43)	0.086
**Household HIV Status** – At least one PLWH vs No PLWH ^b^	0.40(−0.06,0.87)	0.087	0.51(0.04,0.98)	**0.033** *
**Total Study Visits** ^a^		0.284		0.217
1	REF		REF	
2	0.32(−0.80,1.44)	0.571	−0.17(−1.30,0.97)	0.769
3	0.64(−0.24,1.53)	0.153	0.43(−0.45,1.32)	0.335
**Always vs Intermittent shedder (n = 140)**	1.62(1.19,2.05)	**< 0.001** *	--	
**Number of Visits with KSHV Shedding**		**< 0.001** *		
1	REF		--	
2	1.04(0.58,1.50)	**< 0.001** *		
3	2.01(1.57,2.44)	**< 0.001** *		

* p < 0.05 considered statistically significant

All models included a random intercept and random slope for household.

a Adjusted for Age (categorical)

b Adjusted for number household members

c Adjusted for household HIV status (any vs none)

## Abbreviations

KSHV: Kaposi’s sarcoma-associated herpesvirus
KS: Kaposi’s sarcoma
DNA: Deoxyribonucleic acid
PLWH: people living with HIV
GPC: general population cohort
UVRI-REC: Uganda Virus Research Institute Research Ethics Committee
UNCST: Uganda National Council for Science and Technology
COMIRB: Colorado Multiple Institutional Review Board
ART: anti-retroviral therapy
HIV: human immunodeficiency viruses
ORF: Open reading frame
MFI: Median fluorescence intensity
EBV: Epstein-Barr Virus
PBMC: peripheral blood mononuclear cells
ERV-3: human endogenous retrovirus 3
ICC: intraclass correlation coefficient
IDW: Inverse distance weighted
